# Engineering the 3D structure of organoids

**DOI:** 10.1016/j.stemcr.2024.11.009

**Published:** 2024-12-19

**Authors:** Samuel P. Moss, Ezgi Bakirci, Adam W. Feinberg

**Affiliations:** 1 Department of Biomedical Engineering, Carnegie Mellon University, Pittsburgh, PA, USA; 2 Department of Materials Science and Engineering, Carnegie Mellon University, Pittsburgh, PA, USA

**Keywords:** organoids, 3D bioprinting, biofabrication, spheroids, stem cells, vascularization

## Abstract

Organoids form through the sel f-organizing capabilities of stem cells to produce a variety of differentiated cell and tissue types. Most organoid models, however, are limited in terms of the structure and function of the tissues that form, in part because it is difficult to regulate the cell type, arrangement, and cell-cell/cell-matrix interactions within these systems. In this article, we will discuss the engineering approaches to generate more complex organoids with improved function and translational relevance, as well as their advantages and disadvantages. Additionally, we will explore how biofabrication strategies can manipulate the cell composition, 3D organization, and scale-up of organoids, thus improving their utility for disease modeling, drug screening, and regenerative medicine applications.

## Graphical abstract

**Figure:**
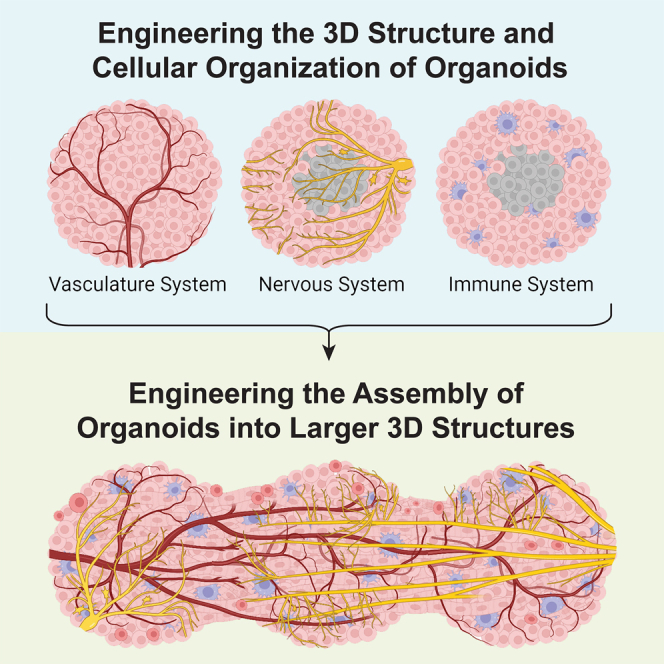

## Teaser

Feinberg and colleagues review approaches to engineer the structure and function of organoids and multi-organoid assemblies to improve translational relevance. They examine strategies focusing on cell composition, 3D organization, and scalability. Furthermore, emerging approaches including 3D bioprinting, organoid-on-chip systems, and machine learning and artificial intelligence and their impact on disease modeling, drug screening, and regenerative medicine using organoids are discussed.

## Introduction

Organoids have emerged as a powerful way to study early tissue and organ development, transitioning from two-dimensional (2D) to three-dimensional (3D) culture conditions that recapitulate key aspects of embryonic morphogenesis. Key to this is cell-mediated self-assembly to form relatively complex 3D structures *in vitro*, typically driven by stem cell specification and differentiation into specific cell and tissue types. This has made organoids a rapidly growing research tool, from basic science studies in developmental biology to applied studies in drug discovery, cell therapies, and regenerative medicine (Arjmand et al., 2023; Clevers, 2016; Fatehullah et al., 2016; Huch et al., 2017; Lancaster and Knoblich, 2014; Miranda et al., 2018; Takahashi, 2019). However, despite the many advances that have been made, the size and complexity of organoids reach a plateau due to limitations of the self-organization process *in vitro*, such as the lack of organism-scale mechanical forces and little to no development of vasculature to provide adequate nutrient and oxygen transport to the growing tissues. To address this, a number of biofabrication techniques have been developed to guide the structure and function of organoids, including 3D bioprinting, microfabrication, and organ-on-chip technologies (Gjorevski et al., 2022; Park et al., 2019; Ren et al., 2021). In this review, we will cover current methods to engineer organoids that move beyond simple self-assembly as well as provide a perspective on future approaches to generate organoids that better recapitulate the structure, function, and phenotype of their *in vivo* tissue counterparts.

## Organoids as a model of tissue and organ development

Organoids are generally defined as multicellular systems produced *in vitro* that self-organize, contain tissue-specific cell types, and display some cytoarchitectural and functional characteristics resembling an organ or tissue. Most organoids are created by first forming spheroids from adult or pluripotent stem cells in hanging drops, microwells, or other similar culture devices that allow the cells to aggregate (Brickman and Serup, 2017). These stem cell spheroids, or embryoid bodies, are then guided to differentiate into organoids, with the specific processes depending on whether the cells are obtained from embryonic stem cells (ESCs), induced pluripotent stem cells (iPSCs), or primary tissues (Pașca et al., 2022; Simunovic and Brivanlou, 2017). However, similar sized cell spheroids and aggregates can also be formed from already differentiated cells to form microtissues that share many of the same properties and methods as used for organoids (Sakalem et al., 2021). Thus, while it is important to understand that organoids and cell spheroids are not the same, there are many core engineering principles that are shared. For this reason, this review discusses the organoid field in a wider sense to include tissue models of similar size such as spheroids and microtissues as well as multi-organoid systems such as assembloids and organ building blocks (OBBs).

**Figure 1:**
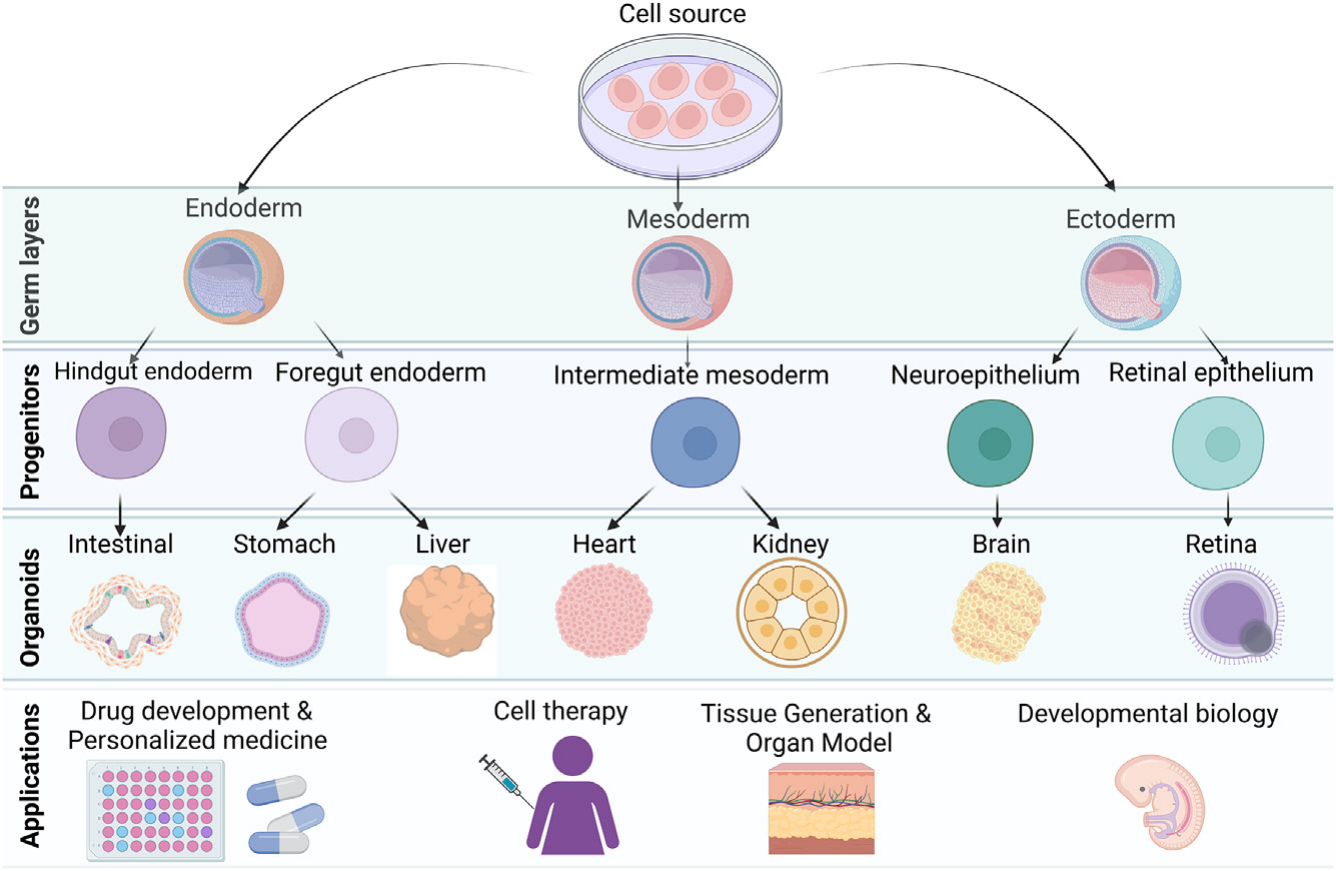
Organoids as a model of tissue and organ development Illustration of organoid generation from pluripotent stem cells. Depending on the protocols, pluripotent stem cells can generate different organoids such as intestinal, stomach, liver, heart, kidney, and brain organoids (Eicher et al., 2022; Hoang et al., 2018; Jin et al., 2018; Lancaster et al., 2013; Lawlor et al., 2021; Sato et al., 2011). Organoids can be used for basic science, disease modeling, drug development, cell therapy, tissue engineering, and regenerative medicine applications. This figure was created with BioRender.com.

The process of organ morphogenesis during embryonic development, in which cells differentiate and self-organize into functional tissues and organs, serves as the basis for creating tissue-specific organoids (Hofer and Lutolf, 2021). This organoid formation is controlled through multiple factors, including intracellular signaling, interactions with neighboring cells, and a combination of biochemical and biophysical factors in the extracellular environment, all of which act in concert to drive cell fate and tissue patterning processes (Huycke and Gartner, 2022). Biomaterials and bioreactors can be used to control the growth and maintenance of organoids by providing the necessary stimuli for cell proliferation, differentiation, and tissue formation. For example, Matrigel, alginate, fibrin, collagen, and polyethylene glycol (PEG) are hydrogels that have been used to modulate the physical microenvironment and control organoid architecture through tunable properties, such as swelling, degradation, and elastic modulus (Broguiere et al., 2018; Capeling et al., 2019; Gjorevski and Lutolf, 2017; Sato et al., 2009). Bioreactors provide essential nutrients and oxygen, improving the growth of large, complex organoids. Together, biomaterials and bioreactors have enabled the development of highly complex systems to generate and grow organoids for a wide range of applications, including disease modeling, drug screening, and regenerative medicine (Brickman and Serup, 2017; Broguiere et al., 2018; Capeling et al., 2019; Hofer and Lutolf, 2021; Park et al., 2019; Pașca et al., 2022; Sato et al., 2009). Numerous different types of organoids have been developed by utilizing these methods to create models of tissues such as the intestine, brain, kidney, and pancreas (Figure 1) (Birey and Sergiu, 2022; Dutta et al., 2017; Kim et al., 2023; Lancaster et al., 2013; Liu et al., 2020a; Nikolaev et al., 2020; Nishinakamura, 2019). These 3D organoid models offer unique opportunities to study developmental processes and model human disease.

## Current limitation of organoids

**Figure 2:**
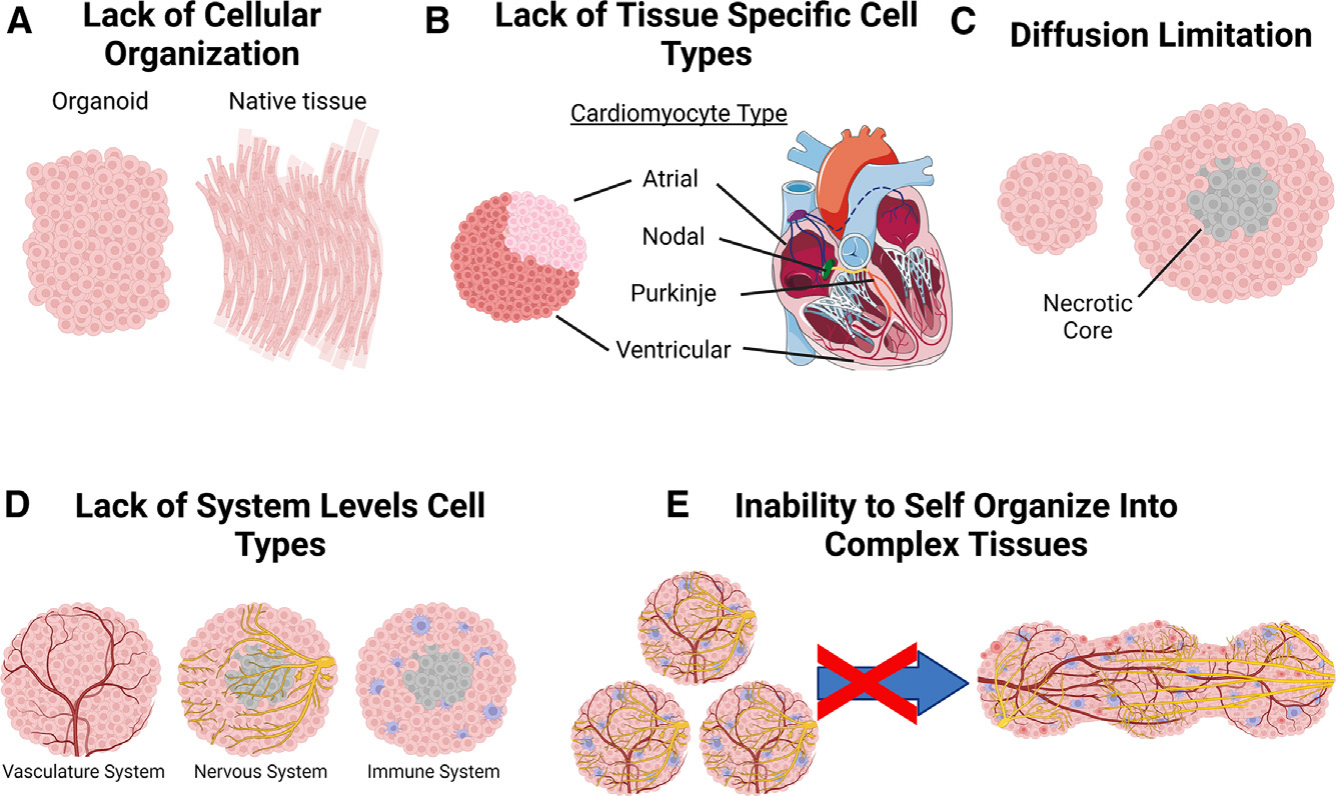
Current limitations of organoids (A) Cells often lack structural cues to organize within an organoid in the same manner as in native tissue, depicted here with poorly organized cardiomyocytes in a cardiac organoid versus highly aligned cardiomyocytes in native heart muscle. (B) Although organoids possess multiple cell types, the recapitulation of all cell types from a tissue is not present in organoids. For example, atrial and ventricular cardiomyocytes are typically found in organoids, but not nodal, Purkinje, and other cell types in the heart. (C) Diffusion of oxygen, nutrients, and waste limits the size in which organoids can form without having a necrotic core. (D) Organoids are also often missing key cell types that span across tissue and organ types, such as the vasculature, nervous, and immune systems. (E) Organoids do not have the ability to self-assemble into complex tissue constructs, which would be required to recapitulate the functional repertoire of native tissue. This figure was created with BioRender.com.

Organoids have enabled many scientific advances; however, there is still a large difference between the cellular composition and structure of organs, as compared to what can be recreated *in vitro* using organoids (Park et al., 2019; Rossi et al., 2018). For example, in cardiac organoids cardiomyocytes typically have a more rounded morphology and random spatial organization, while in native cardiac tissue cardiomyocytes are elongated and highly aligned (Figure 2A). This is important because cardiomyocyte alignment has been shown to lead toward functional maturation of cardiac tissues (Carson et al., 2016; Kim et al., 2010). Additionally, organoids typically lack the entire range of cell types found in native tissue. For instance, cardiac organoids may possess atrial and ventricular cardiomyocytes, but *in vivo* there are additional neural, immune, stromal, and vasculature cell types as well as cardiomyocyte subtypes including nodal and Purkinje cells and specification for the left and right sides of the heart (Figure 2B) (Devalla and Passier, 2018; Lee et al., 2020). Size is another issue because as organoids proliferate and grow, they are limited in size by the diffusion of nutrients, oxygen, and metabolic waste. When diffusion can no longer achieve proper transport, a necrotic core will occur (Figure 2C) (Xiang et al., 2017). Necrotic core development leads to a decrease in cell viability as well as variable cell differentiation and patterning between inner and outer layers of cells (Grebenyuk and Ranga, 2019; Zhang et al., 2021). The lack of a functional vascular system is a major cause of the limited diffusion of nutrients, oxygen, and waste products, and signaling between the vasculature and surrounding cell types can be critical for physiological function (Takebe et al., 2013). Indeed, organoids are typically missing cell populations and structures that span across tissues and organs, including the immune, nervous, and vascular systems (Figure 2D) (Birey et al., 2017; Chen et al., 2020; Jun et al., 2019). The relatively small size of organoids limits the complexity these systems can achieve, and organoids lack the ability to self-organize into larger-scale constructs that would be required to achieve further structure and function (Figure 2E). Finally, there can be issues with reproducible fabrication of organoids due to variability within and between protocols in terms of shape, structure, and function. Additional discussion on the limitations of organoids can be found here (Andrews and Kriegstein, 2022; Hofer and Lutolf, 2021).

## Approaches to engineer organoids with improved structure and function

To address current limitations, organoid engineering—the application of bioengineering principles to create more complex and functional organoids—has seen a number of advances (Xinaris et al., 2015; Yin et al., 2016). This has allowed for modeling anatomic and physiologic aspects of the kidney, brain, and intestine that are difficult to achieve with traditional organoid culture. Here we define organoid engineering to include organoid-on-chip, microfabrication, and 3D bioprinting to structure and organize the organoids in a more defined way and provide environmental cues such as nutrient gradients or mechanical stimuli. By providing a more physiologically relevant context, these approaches can help to drive more complex and reproducible organoid culture systems.

### Organoid-on-chip systems

**Figure 3:**
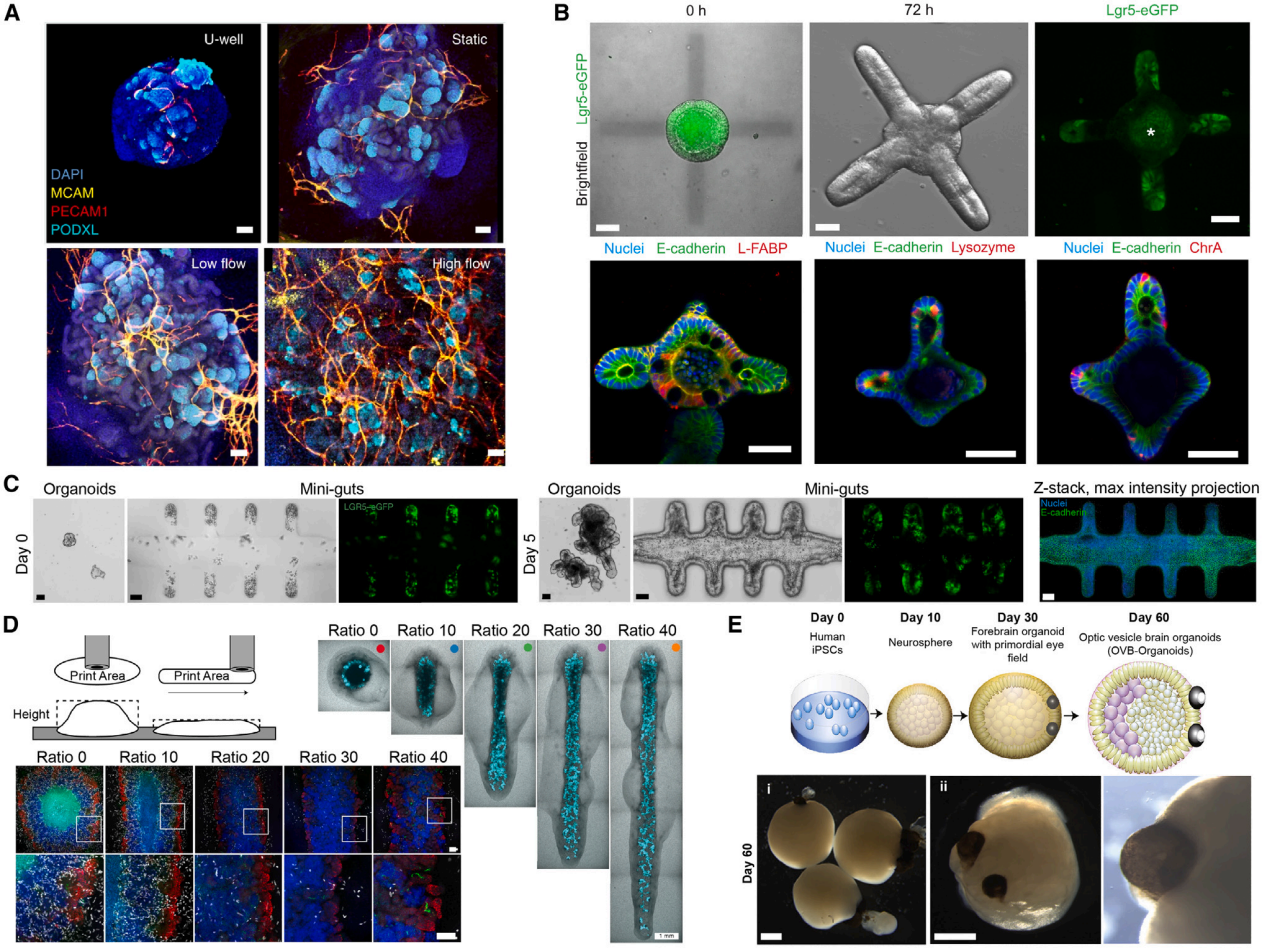
Engineering structure at the organoid scale (A) Vascular markers in kidney organoids cultured under static U-well, static engineered ECM, low-shear-stress, and high-shear-stress conditions. Scale bars are 100 μm (Homan et al., 2019). (B) Intestinal organoids in a photo-responsive PEG-based hydrogel that exhibits a decreased Young’s modulus when subjected to 405 nm light. Cells migrate into the softened region within 72 h with Lgr5+ cells largely localized in the tips of the protrusions. Fluorescent staining depicts nuclei (blue), cell-cell junctions (green), and L-FABP, lysozyme, ChrA (red, from left to right). Scale bars are 30 μm (Gjorevski et al., 2022). (C) Intestinal stem cells seeded into a laser-ablated channel in a Matrigel-based hydrogel, which proliferate into a perfusable intestinal organoid with a lifespan of at least one month. Scale bars are 50 μm (Nikolaev et al., 2020). (D) Kidney organoids were printed with varying shapes from single spots to 12 mm lines, which affects the development of various nephron structures. All printed organoids possess approximately the same number of cells. Kidney organoids were stained for MAFB^mTagBFP2^ (blue), SOX17 (gray), SLC12A1 (green), and HNF4a (red). Scale bars are 100 μm (Lawlor et al., 2021). (E) Schematic depicting the overall steps of the formation of optic vesicle containing brain organoids (top). Day 60 brain organoids with pigmented optical vesicles and individual pigmented optic vesicle (bottom) are shown. Scale bars are 1 mm (Gabriel et al., 2021).

Organoid-on-chip systems combine organoids and microfluidics into integrated devices to create a biomimetic and dynamic *in vitro* microenvironment that better recapitulates physiologic conditions. To do this, these organoid-on-chip systems use micropatterning of biochemical factors, mechanical loading, electrical stimulation, soluble biochemical gradients, and fluid flow, alone or in combination, to better mimic tissue and organ function (Bhatia and Ingber, 2014). For example, fluid shear stress can stimulate the development of microvascular networks with perfusable lumens and when applied to renal organoid-on-chip systems leads to tissue maturation and morphogenesis including the formation of proximal tubule and glomerular compartments (Figure 3A) (Homan et al., 2019). Perfusion and fluid shear stress have also been shown to improve functional maturation of pancreatic, neural, and intestine organoid-on-chip systems (Cho et al., 2021; Tao et al., 2019; Workman et al., 2018; Zhang et al., 2021). Further, the addition of a peristaltic pump to generate cyclical pressure changes combined with perfusion and fluid shear stress can more accurately mimic the *in vivo* environment of the stomach. This is very useful for maintaining long term organoid cultures because it replicates the dynamic mechanical environment present in stomach (Lee et al., 2018). For example, Fang et al. designed a colon tumor organoid on chip with a pressure channel that mimicked intestinal peristalsis where the organoids contracted periodically and exhibited reduced uptake of ellipticine-loaded micelles as a model of drug resistance (Fang et al., 2021). Additional capabilities include electrical stimulation of cardiac spheroids and organoids to improve maturation and real-time measurement of O_2_ consumption to assess metabolic function (Gu et al., 2024; Liu et al., 2020b). In summary, by tailoring the biochemical, biomechanical, and electrical conditions to meet the specific needs of different organoids, the organoid-on-chip technology can better mimic physiologic conditions for a broader range of research questions (Fang et al., 2023; Zhao et al., 2024).

### Engineering geometric constraints through microfabrication

Microfabrication can be used to engineer organoids by controlling physical and geometric constraints in the microenvironment that directly affect cell behavior. For example, cell viability was improved in hepatic spheroids by microfabrication into elliptical tubes termed cylindroids (Fukuda et al., 2004). This effectively increased the surface area-to-volume ratio, which minimized the necrotic core due to a larger proportion of the cells being near the spheroid surface and within the diffusion limit of oxygen and nutrients. A similar result was found through seeding a mixture of rat hepatocellular carcinoma cells and human fibroblasts together into rods, tori, and honeycomb patterns, which similarly minimized necrotic cores by reducing diffusion distances (Dean et al., 2007). Geometric constraints have also been applied to control cell alignment within organoids. Huebsch et al. seeded cardiomyocytes within dog bone-shaped polydimethylsiloxane molds and achieved alignment of cardiomyocytes in the linear region of the mold, which resulted in an increased conduction velocity (Huebsch et al., 2016). Similarly, Karzbrun et al. were able to model human neural tube morphogenesis by first constraining pluripotent stem cells to 2D cultures in small lines with widths ranging from 150 to 1,500 μm and then transitioning to 3D culture in Matrigel (Karzbrun et al., 2021). Switching to neural induction medium caused the cells to form a neural tube-like structure that was able to recapitulate morphogenetic events such as the development of the human amnion, forebrain, and dorsoventral axis. In terms of intestinal organoids, Lutolf and co-workers have demonstrated the ability to control cell patterning and morphogenesis in multiple ways (Gjorevski et al., 2022). They first seeded intestinal stem cells (ISCs) into photosensitive PEG-based hydrogel, where patterned photodegradation led to ISC infiltration with localized Lgr5^+^ cells and crypt-like budding (Figure 3B), where the Lgr5 gene is used as an ISC marker (Barker et al., 2007). The group also used soft lithography to pattern rod-shaped cavities into collagen type I and Matrigel hydrogels that were seeded with ISCs to form aggregates where Lgr5^+^ cells and crypt-like buds localized to the rod ends. In the most complex example, a collagen and Matrigel hydrogel was laser ablated to yield a channel with crypt-like microcavities and seeded with ISCs that formed a confluent layer, and, eventually, Lgr5 expression was localized within the crypt-like structures (Figure 3C) (Nikolaev et al., 2020). The intestinal surface was able to shed cells and relieve the pressure created from proliferative overcrowding that occurs in traditional lumenized organoids. The hydrogel was integrated into a perfusable system that allowed for continuous flow throughout the channel and removal of dead cells from the shedding process. Typically, this accumulation of dead cells results in intestinal organoids having a lifespan of approximately 10 days, but, in this system, the lifespan was extended to at least one month. Geometric constraints to localize crypt-like microcavities have also been fabricated using 2 photon lithography to crosslink photosensitive gelatin and PEG in Matrigel (Urciuolo et al., 2023). The photosensitive polymers diffused into Matrigel containing mouse small intestine organoids before crosslinking with 2 photon lithography to define stiffer regions encompassing individual organoids. The defined geometry included 3D microcavity spaces, which the organoids grew into and over time LGR5 expression was localized to. This study also performed similar experiments to control apical/basal polarization in human fetal hepatocyte organoids and tip budding in mouse fetal lung rudiments. In these examples, the geometries in which cells were initially constrained were able to control certain cellular aspects such as cardiomyocyte alignment, neural tube morphogenesis, and the localization of crypt-like structures.

### Bioprinting at the organoid scale

3D bioprinting of organoids is a robotic biofabrication process that has the advantages of automation and repeatability and can assemble organoids into more complex 3D structures (Galliger et al., 2019; Mazzocchi et al., 2019; Murphy and Atala, 2014; Vijayavenkataraman et al., 2018; Zhang et al., 2017a). The most common bioprinting approaches for structured organoid biofabrication are summarized in Table 1.

**Table 1:** Bioprinting methods to engineer organoids with improved function and structure at both the organoid scale and organizing organoids relative to one another

Bioprinting method	Benefits	Main limitations	Demonstrated tissue types
Extrusion	3D placement of cells and/or whole organoids. Easy to manufacture heterogeneous constructs using multi-printhead bioprinters.	extrusion shear stress may affect cell viability limited 3D geometries without embedding bioink requires specific rheological properties for printing	kidney (Lawlor et al., 2021), stomach/intestine (Brassard et al., 2020) vasculature (Norotte et al., 2009), colon (Töpfer et al., 2019), mammary epithelium (Reid et al., 2018), Cartilage tissue (Decarli et al., 2023; Moor et al., 2020)
Extrusion – coaxial	fastest method tube-like structures (e.g., vasculature)	limited material compatibility and geometries	cardiac (Zhang et al., 2017b), vasculature (Jia et al., 2016; Shao et al., 2020)
Extrusion – embedded	fabricate vascular-like networks in multi-organoid structures (e.g., SWIFT)	requires a large number of organoids geometries limited by molds	cardiac (Skylar-Scott et al., 2019; Stankey et al., 2024),
Pick and place	accurate positioning of single organoids	slower than other printing processes challenging to manufacture complex geometries limited scale-up potential	smooth muscle (Itoh et al., 2015), cartilage, bone, cardiac (Moldovan et al., 2017), cartilage and bone (Ayan et al., 2020b), cardiac (Daly et al., 2021), neural and cancer (Roth et al., 2023), liver (Ip et al., 2018)
Volumetric	light-based approach faster than other printing processes forms entire 3D structure at one time	potential UV cytotoxicity requires photocrosslinkable materials light scattering from cells limits the resolution	liver (Bernal et al., 2022),

The 3-axis Cartesian robotic system typically used for 3D bioprinting can achieve precise placement of cells in a repeatable manner for organoid and spheroid fabrication. For example, Lawlor et al. printed kidney organoids onto Transwell membranes ranging from single spots to 12 mm lines, while keeping the number of cells constant in all conditions (Lawlor et al., 2021). The linear organoids had a high number of nephrons and upregulation of genes associated with mature tubule function (Figure 3D), showing how small changes in printed geometry can affect how cells self-organize, form more complex structures, and alter gene expression. In this case, it is important to note that the change in geometry also reduced organoid thickness, potentially preventing necrotic core formation and leading to improved morphogenesis. At a larger scale, mouse ISCs printed into dense lines within both Matrigel and stiffer Matrigel/collagen hydrogels developed into long lumenized intestinal organoids in an embedded printing process termed bioprinting-assisted tissue emergence (BATE) (Brassard et al., 2020). These tubular organoids show cellular patterning and organization of Paneth cells and stem cells similar to what is found in the intestinal crypts *in vivo* but are limited in how long they can be cultured as dead cells are shed into the lumen. However, they can be cultured for longer than classical intestinal organoids (≥3 weeks) and better resemble the phenotype and functionalities of their *in vivo* counterparts. BATE was also used to print a gradient of mouse stem cells from the stomach, small intestine, and colon that formed a large-scale organoid from stomach corpus on one end to small intestine at the other. These approaches show that printing linear cells aggregates can enhance the formation of tubular organoids.

Bioprinting has also been used to introduce rudimentary vasculature networks into cell aggregates and organoids. Airflow-assisted 3D bioprinting was used to create spiralized patterns of human umbilical vein endothelial cell (HUVEC) networks within mesenchymal stem cell (MSC) spheroids, which when cultured in osteogenic media formed vascularized bone spheroids (Zhao et al., 2018). Coaxial extrusion 3D bioprinting was used to fabricate grid structures of gelatin methacryloyl (GelMA) and alginate with HUVECs suspended in the mixture on to which other cells such as cardiomyocytes can be seeded form prevascularized spheroids (Zhang et al., 2017b). A similar result using a printed HUVEC network was demonstrated with the addition of PEG-tetra-acrylate to the bioink to tune rheological and mechanical properties (Jia et al., 2016). All together, these bioprinting methods leverage the ability to precisely position cells for organoid and spheroid fabrication to generate prevascular and vascular-like networks, with more comprehensive review on variation of these methods available (Grebenyuk and Ranga, 2019).

### Additional approaches for engineering at the organoid scale

There are additional methods that have been developed to control cellular organization, expand cellular heterogeneity, and increase structural complexity of organoids. For example, Wijesekara et al. reversed the polarity of airway organoids by aggregating human airway basal stem cells together without any extracellular matrix (ECM) support to have the cilia covered, apical membrane facing outward to facilitate functional studies (Wijesekara et al., 2022). Chemical factors are also widely used to drive specific cell responses during organoid formation. Retinoic acid is critical for early eye development and when applied to differentiating neurospheres can form entire optic vesicles with primitive corneal epithelial and lens-like cells, retinal pigment epithelia, retinal progenitor cells, axon-like projections, and electrically active networks within brain organoids (Figure 3E) (Gabriel et al., 2021). Molidustat is a small molecule that upregulates the hypoxia-inducible factor pathway, which is upstream of vascular endothelial growth factor and has been used to stimulate prevascularization of cardiac spheroids (Coyle et al., 2021). Vascularization within organoids has also been induced using genetic engineering approaches. Lentiviral transduction in human iPSCs to alter specific gene regulatory networks was used to create designer liver organoids that developed an improved vasculature network compared to fetal liver organoids based on vessel length, density, genes expression, and function *in vitro* (Velazquez et al., 2021). Vascular networks can also be formed directly, as demonstrated by Strobel et al. who combined isolated microvessel fragments with MSCs to create vascular networks within adipose-like organoids (Strobel et al., 2021). These are a few examples of the multiple approaches that can be used to engineer the structure and function of organoids beyond what is possible with self-assembly alone.

## Engineering the interactions between organoids to build at larger scales

The ability to engineer structured organoids with improved function also provides opportunities to combine these organoids together as building blocks in larger-scale constructs. The approaches that have been developed to build single organoids can also be adapted to combine multiple organoids together and direct how they interact and fuse together. These engineering methods might control the spatial arrangement of organoids for the generation of larger tissue-like constructs that can better recapitulate some of the physiology of native tissue.

### Organoid fusion into assembloids

Fused organoids can address one of the main limitations of single organoids, which frequently lack the complex interplay between different tissue types. Assembloids, which are generated by combining different organoids or by including missing cell types into organoids, enable formation of more complex tissue constructs (Kanton and Pas, 2022; Schmidt, 2021). Kim et al. generated bladder assembloids that have an epithelial layer enclosing a stromal layer with a muscular layer on the outside. They exhibited features of adult bladder in terms of cell composition, gene expression, and regenerative response to injury (Kim et al., 2020). Researchers have also combined brain organoids with endothelial and mesenchymal cells, utilizing ets variant transcription factor 2 (ETV-2)-induced endothelial differentiation, and integrating human ESC-derived vascular organoids, to address the challenge of vascularization in organoids and assembloids (Cakir et al., 2019; Song et al., 2019; Sun et al., 2022). Additionally, assembloids have been used to examine tissue interactions. For example, Linkous et al. created assembloids by merging human brain area-specific organoids with glioblastoma spheroids in order to improve current preclinical brain cancer models (Linkous et al., 2019). In another example, Koike et al. showed that endoderm-derived organoids can be promoted by juxtaposing anterior and posterior gut organoids, which together can generate hepato-biliary-pancreatic domains (Koike et al., 2019). Lindberg et al. demonstrated that the degree of fusion and ECM deposition depends on the contact timing relative to the OBB maturation state (Lindberg et al., 2021). Overall, these assembloids hold the potential for modeling complex multi-tissue crosstalk and cellular interactions in multiple disease states.

### Bioprinting to organize organoids

**Figure 4:**
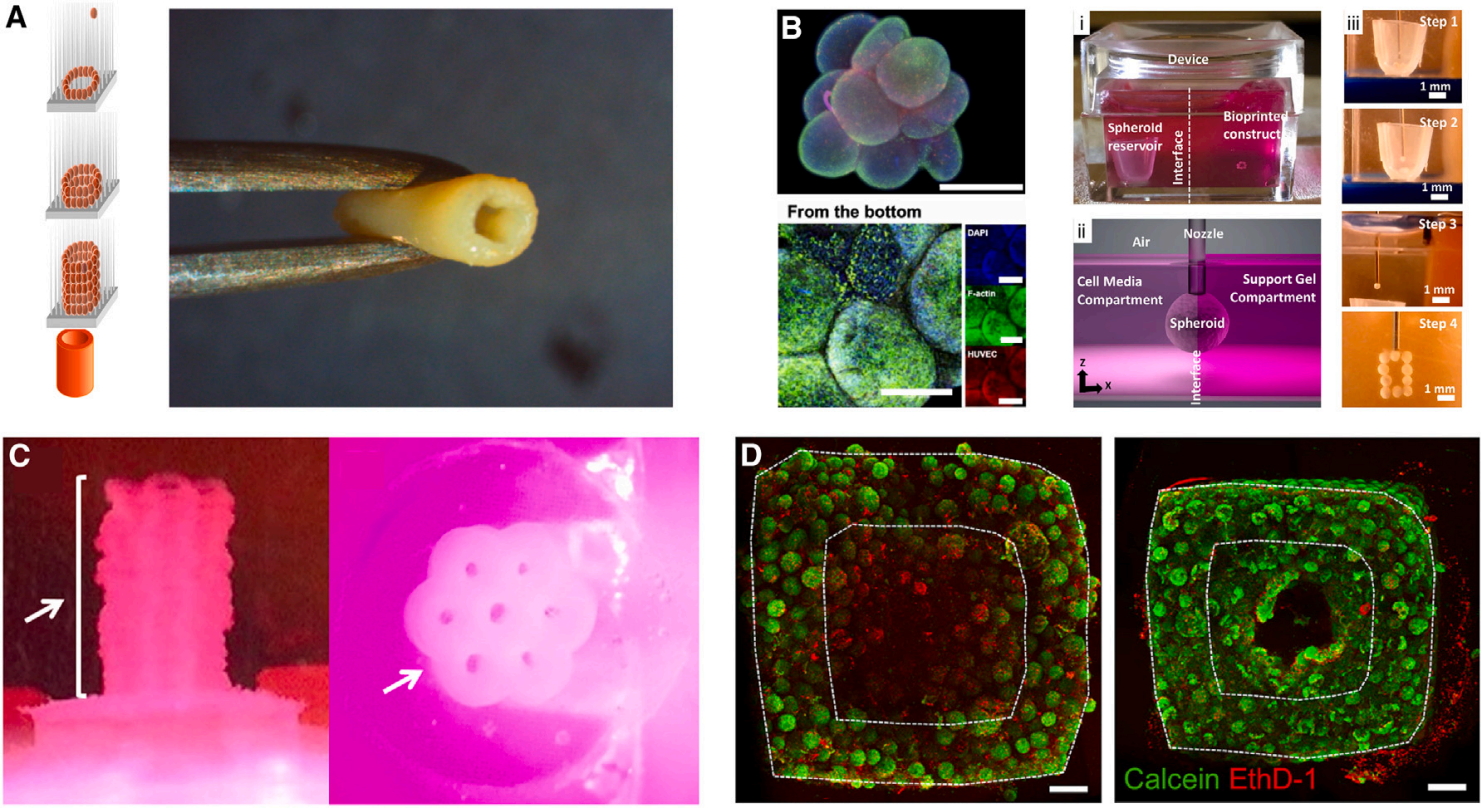
Engineering the interactions between organoids to build tissues (A) The Kenzan 3D bioprinting method places cell spheroids and organoids onto a microneedle area, and then the spheroids fuse together into a tissue construct. Adapted under the terms of the CC-BY license (Itoh et al., 2015). (B) Aspiration-assisted 3D bioprinting of MSC/HUVEC spheroids showing a pyramid of MSC/tdTomato+ HUVEC spheroids (top left). Scale bar is 1,000 μm. Confocal images of the pyramid construct highlight the bottom view (bottom left). Scale bars are 500 μm and 200 μm. Images of the entire device (i), media-gel interface (ii), and overall fabrication procedure (iii) of aspiration-assisted bioprinting (Banerjee et al., 2022). (C) Pick-and-place methods can stack and align twenty honeycomb-shaped microtissues with integrated lumens (Ip et al., 2016). (D) Comparison between organ-specific tissues that were printed with embedded vascular channels (right) and tissues grown without these channels (left). Scale bars are 500 μm (Skylar-Scott et al., 2019).

Bioprinting provides a precise and controlled environment for organoid placement and growth, which enables the production of highly organized and reproducible 3D tissue structures. Extrusion of bioinks containing cell aggregates and organoids has also shown promise. Norotte et al. extruded cellular aggregates in the form of strands to generate vascular trees (Norotte et al., 2009). Topfer et al. used colonoid-laden GelMA bioink and printed it in 96-well plates to generate a multi-well screening platform in combination with in-plate cryopreservation (Töpfer et al., 2019). Similar approaches used spheroid-laden hydrogel bioinks to form 3D cartilage tissue scaffolds (Decarli et al., 2023; Moor et al., 2020). Reid et al. showed that the cell number and position of organoids are crucial factors in the production of large-scale, branched tubular structures of epithelial origin because of their impact on cell-cell interactions (Reid et al., 2018). Another promising method is pick-and-place bioprinting, which enables the precise localization of single organoids relative to one another to form larger tissue constructs after organoids fuse together. For example, the Kenzan method positions spheroids in 3D by impaling them on microneedle arrays that are spaced to enable spheroid fusion into plates, tubes, and heterogeneous tissues (Figure 4A) (Haag et al., 2022; Itoh et al., 2015; Moldovan et al., 2017). More precise manipulation of spheroids and organoids has been demonstrated using aspiration-assisted bioprinting and micromanipulation (Figure 4B) (Ayan et al., 2020a; Heo et al., 2020), where spheroids are printed on matrix-coated substrates or within a self-healing hydrogel support matrix. Ayan et al. showed that precise positioning of spheroids is an effective tool to analyze cell signaling among distinct cell types, such as human dermal fibroblasts in fibrin causing HUVEC spheroids to exhibit angiogenic sprouting (Ayan et al., 2020b). Daly et al. have used a similar technique to simulate post-myocardial infarction scarring by creating localized regions rich in cardiac fibroblast spheroids (Daly et al., 2021). In another approach, Roth et al. developed spheroid transfer assisted by magnetic printing (STAMP), a method for controlling the spatial arrangement of more delicate organoids. STAMP employs a magnetic nanoparticle-laden cellulose nanofiber hydrogel and a magnetized 3D printer (Roth et al., 2023) to arrange neural organoids from human iPSCs and glioma organoids from patients in 3D cancer assembloids. Similarly, Blakely et al. developed the Bio-Pick, Place, and Perfuse (Bio-P3) bioprinting method to form a scalable tissue construct using large-sized spheroids and honeycomb cell aggregates (Blakely et al., 2015). The same group also successfully generated microtissues including 100 million HepG2 cells and then assembled twenty microtissues together (Figure 4C) (Ip et al., 2018). These pick-and-place approaches are useful for studying heterogeneous interactions between different OBBs and for building more complex tissue constructs for drug screening. The major downside of pick-and-place approaches is relatively slow printing time, which limits utility for large-scale biofabrication.

Advances in 3D bioprinting have demonstrated that it is possible to organize organoids into complex structures and to manufacture larger-scale tissues (Wolf et al., 2022). For example, Skylar Scott et al. used an embedded bioprinting technique termed sacrificial writing into functional tissues (SWIFT) for the construction of highly perfusable cardiac tissue by direct printing of vascular channels into a living 3D matrix of densely packed human iPSC-derived cardiac spheroids (Figure 4D) (Skylar-Scott et al., 2019). A synchronously beating tissue was achieved through spheroid fusion to form perfumed and vascularized cardiac tissues. This approach has been further iterated on through the incorporation of a coaxial nozzle to localize smooth muscle cells around the printed vascular channels to increase the physiologic relevance of these vascular-like features (Stankey et al., 2024). In addition, Lie et al., bioprinted neurospheroid-laden alginate, gelatin, GelMA, and laminin bioink into a GelMA support bath with astrocytes and calcium to generate brain-like tissue (Li et al., 2020). To print larger tissues more rapidly, Riccardo et al. used a volumetric bioprinting approach to produce complex constructs containing liver organoids by crosslinking organoid-laden GelMA on a rotating platform. Instead of building the structure layer by layer, they utilized light to project a 3D pattern (volumetric bioprinting) of the entire structure at once from the organoid containing GelMA (Bernal et al., 2022). Overall, though the 3D bioprinting of organoids is still in its infancy, the technology holds promise for the engineering of multi-organoid tissue constructs.

## Future perspectives

This review presents different strategies to engineer the structure of organoids in order to expand the function and physiologic relevance of these systems. Indeed, much of the current research is focused on ways to improve the relevance of organoids as representative models of their *in vivo* tissue and organ counterparts. Many approaches aim to apply a form of external geometric constraint to control structure formation and cellular organization. One can imagine additional ways to do this beyond what has been demonstrated to date, such as incorporating physical cues within the organoids to influence the surrounding cells as they grow and undergo spatial patterning. As organoid design, fabrication, perfusion systems, and culture scale-up continue to progress, it is anticipated that organoids will be used in the future for *de novo* biofabrication of multi-tissue constructs for disease modeling and therapeutic applications (Skardal et al., 2017).

Future research in engineering structured organoids will likely be shaped by three key approaches: 3D bioprinting, organoid-on-chip systems, and artificial intelligence (AI). These technologies aim to create organoids with more mature phenotypes, higher cellular diversity, improved physiologic function, and greater clinical relevance. 3D bioprinting enhances spatial control, allowing the precise positioning of cells or organoids to create complex and larger-scale constructs. This technology facilitates direct fusion of organoids to create multi-organoid systems. Hybrid approaches that combine multiple 3D bioprinting processes together have the potential to produce more complex and hierarchical structures (Dalton et al., 2020). Organoid-on-chip and perfusion bioreactors better mimic *in vivo* conditions, and integrating real-time sensors on these platforms can measure metabolism and hormone secretion and provide additional insights into homeostasis (Norfleet et al., 2020). Machine learning (ML) and AI are revolutionizing the analysis and optimization of experimental outcomes for organoids. These technologies excel in handling complex datasets and identifying patterns for the development of novel diagnostic and therapeutic strategies. For example, ML automates the monitoring of dynamic changes in organoid morphology, number, and size while enhancing detection, classification, and measurement in pharmacological research (Kraus and Frey, 2016; Rojas-Moraleda et al., 2017). Additionally, ML can tailor organoid experiments for personalized medicine by analyzing patient-specific genomic data (Matthews et al., 2022; Monzel et al., 2020; Spiller et al., 2021; Zhou et al., 2023). It is expected that 3D bioprinting, organoid on chip, and AI/ML will be synergistic, working together to improve control of the microenvironment and replicate physiologic conditions that lead to better cellular maturation and improved organoid function.

In summary, despite key challenges, organoid technologies have been advancing and will continue to do so by incorporating research including stem cell science, bioengineering, biomaterials, biofabrication, biophysics, and computer science. It has made significant strides in translation toward preclinical and clinical applications with studies demonstrating the potential of organoids in personalized medicine settings, drug discovery, regenerative medicine, and gene therapy (LeSavage et al., 2022; Lou and Leung, 2018). Nonetheless, progress is hindered by various challenges, such as creating organoids with consistent geometries and cell organization as well as extending their lifespan to yield mature, functional tissues. Advancing the development of structured organoids requires multidisciplinary approaches to progress organoids toward models and building blocks of functional tissues and organs.

## Acknowledgments

A.W.F. received funding from the Additional Ventures Foundation through the Cures Collaborative and the Breakthrough T1D Foundation (2-SRA-2021-1024-S-B).

## Author contributions

S.P.M. and E.B. compiled literature research, wrote the manuscript, and produced the figures. A.W.F. conceptualized, reviewed, and edited the manuscript. All authors read and approved the final manuscript as submitted.

## Declaration of interests

A.F. has an equity stake and is the Chief Technology Officer in FluidForm Inc., a startup company commercializing FRESH 3D bioprinting.
